# Molecular dynamics simulation of carbon nanotube growth under a tensile strain

**DOI:** 10.1038/s41598-024-56244-6

**Published:** 2024-03-07

**Authors:** Ayaka Yamanaka, Ryota Jono, Syogo Tejima, Jun-ichi Fujita

**Affiliations:** 1 Research Organization for Information Science and Technology, 7F, Sumitomo-Hamamatsucho Building, 1-18-16, Hamamatsucho, Minato-ku, Tokyo, 105-0013 Japan; 2 Graduate School of Pure and Applied Science, University of Tsukuba, 1-1-1 Ten-nodai, Tsukuba, Ibaraki 305-8573 Japan

**Keywords:** Carbon nanotubes and fullerenes, Chemical physics

## Abstract

We performed molecular dynamics simulations of carbon nanotube (CNT) to elucidate the growth process in the floating catalyst chemical vapor deposition method (FCCVD). FCCVD has two features: a nanometer-sized cementite (Fe_3_C) particle whose melting point is depressed because of the larger surface-to-volume ratio and tensile strain between the growing CNT and the catalyst. The simulations, including these effects, demonstrated that the number of 6-membered rings of the (6,4) chiral CNT constantly increased at a speed of ${1}\,{\textrm{mm}/\textrm{s}}$ at ${1273}\,{\textrm{K}}$, whereas those of the armchair and zigzag CNTs were stopped in the simulations and only reached half of the numbers for chiral CNT. Both the temperature and CNT chirality significantly affected CNT growth under tensile strain.

## Introduction

Carbon nanotubes (CNTs) are one-dimensional carbon allotropes comprising sp^2^ carbon networks with unique properties^1–4^. Based on their unique structure, which is a rolled-up two-dimensional graphene to cylinder, they have many favorable characteristics, including mechanical strength, and thermal and electrical conductivity. CNTs have attracted much attention of many researchers since their synthesis^5^ and identification^6^ due to these properties. For example, the tensile strength of CNTs is more than ${80}\,\textrm{GPa}$^7^, while that of stainless steel is only ${1}\,\textrm{GPa}$^8^, and the thermal conductivity of CNTs is ${6000}\,\text{W}/\text{m K}$, while that of copper metal is ${500}\,\text{W}/\text{m K}$^9^. Various synthesis methods have been developed so far, including laser ablation^10^, arc discharge^11^, and chemical vapor deposition (CVD)^12–14^. Substrate-based CVD is widely used because of its low-cost and scalable methods for mass production of CNTs. Another type of the CVD, floating catalyst CVD (FCCVD) have also attracted many researchers because of its high-speed CNT growth^15–21^. The reported growth speed of CNT in FCCVD is from 0.1 to 100 μm/s^22,23^ and sometimes reaches ${1}\,\textrm{mm}/\textrm{s}$^24^, and is faster than that in substrate-based CVD whose speed is from ${1}\,\textrm{nm}/\textrm{s}$ to 10 μm/s^25,26^. The CNT grows on the flying nm size cementite (Fe_3_C) particles in the FCCVD method, while it grows on a substrate in substrate-based CVD. The dynamics of carbon in cementite nanoparticles are faster than those on the substrate because of the melting-point depression from the larger surface-to-volume ratio, and the growing CNT may be pulled and feel tensile strain by the interaction with flying nanoparticles in the FCCVD process. It is necessary to elucidate these effects on the high-speed growth mechanism of CNT in the FCCVD method for more efficient production of CNT; however, there are no experimental reports owing to the difficulty in directly observing the CNT growth progress using floating catalysts. Molecular dynamics (MD) simulations are powerful methods for investigating the growth of CNTs at the atomic level. Many MD simulations of CNT growth have been reported thus far. However, most of these studies involved CNT formation via natural deposition on saturated carbon atoms from the catalyst^27,28^. In this study, we calculated the dynamics of carbon in cementite nanoparticles and introduced tensile strain to the CNT growth simulation to reveal its effects on CNT growth in the FCCVD process.

## Methods

**Figure 1:**
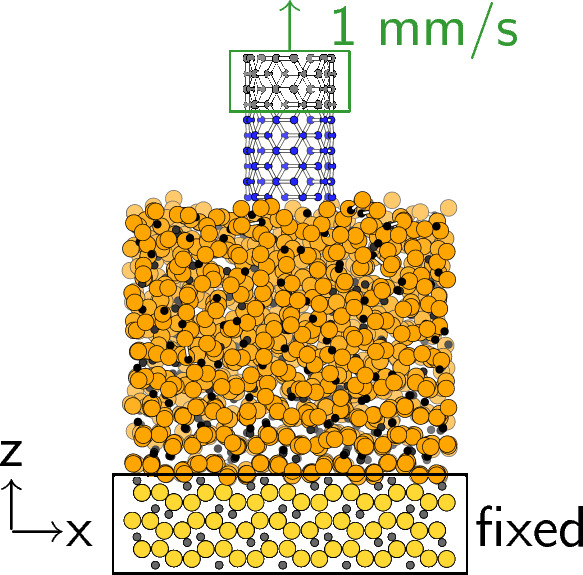
Simulation models of the CNT on cementite (Fe_3_C).

We focused on the CNT-catalyst interface and modeled the initial stage of CNT growth on a cementite (Fe_3_C) (001) surface, as shown in Fig. 1. The sizes of the simulation boxes were determined from the reported structure^29^ as ${2.54}\,\times \, {2.61}\,\times {8.00}\,\textrm{nm}$. The bottom of the ${1}\,\textrm{nm}$ layers for cementite was fixed in space. To obtain the equilibrium state of the cementite structure at ${1073}\,\textrm{K}$, ${1273}\,\textrm{K}$, and ${1473}\,\textrm{K}$, which are below, above, and much above the melting point of cementite, respectively, we performed the NVT simulations under Nosé-Hoover thermostat^30–32^ for ${100}\,\textrm{ns}$. The positions and velocities are time-integrated by using velocity Verlet method with ${1.0}\,\textrm{fs}$ for time step. To introduce the tensile strain, the top ${0.4}\,\textrm{nm}$ of the CNT was pulled up at a constant velocity during the simulations, which was set to ${1}\,\textrm{mm}/\textrm{s}$ to attempt the experimentally reported upper limit of the growth speed^24^. The total MD simulation time was ${500}\,\textrm{ns}$, *i.e.*, the CNT was pulled up for ${0.5}\,\textrm{nm}$, which is correspond to the length of the two 6-membered rings. We considered CNTs with diameters of ${0.7}\,\textrm{nm}$ and the chirality of the armchair (5,5), chiral (6,4), and zigzag (9,0) CNTs to compare the differences in the growth processes depending on the CNT structure. All calculations were performed using the LAMMPS package^33^. There are many kinds of force fields for the mixture of iron and carbon systems, but almost all of them are not suitable for simulating the cementite structure, which is considered as the basic structure in the high-temperature FCCVD process. The previous study reported that only the embedded atom method (EAM) by Lau et al.^34^ and Ruda et al.^35^ and the short-range Tersoff-Brenner-type analytical bond order potential (ABOP) by Henriksson et al.^29^ are the reliable potentials to describe the properties of cementite^36^. Among them, Henriksson’s ABOP can reproduce the 6-membered ring structure of carbon allotropes^37^ because it uses Brenner’s reactive bond-order (REBO) potential^38^ for carbon-carbon interaction, whose accuracy is well tested by comparison with density functional theory. It should be noted that the development of ReaxFF to simulate the mixture of iron and carbon system is continuing, but the simulation by ReaxFF potential takes much longer time than that by ABOP. Based on these references and our preliminary tests, we used the Henriksson’s ABOP to simultaneously describe the interaction of atoms in the cementite and CNT in this work.

## Results and discussions

**Figure 2:**
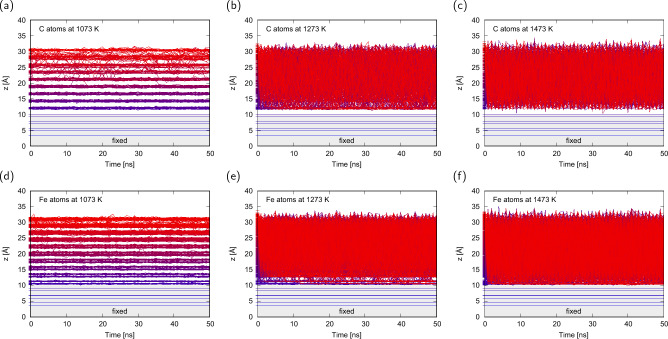
Z position of the (**a**, **b**, **c**) carbon and (**d**, **e**, **f**) iron atoms in cementite at (**a**, **d**) ${1073}\,\textrm{K}$, (**b**, **e**) ${1273}\,\textrm{K}$, and (**c**, **f**) ${1473}\,\textrm{K}$ during the last ${50}\,\textrm{ns}$ of ${100}\,\textrm{ns}$ equilibration. The colors from blue to red correspond to the z position at ${50}\,\textrm{ns}$.

Figure 2 shows the z-oriented trajectories of carbon and iron atoms in the cementite nanostructure at ${1073}\,\textrm{K}$, ${1273}\,\textrm{K}$, and ${1473}\,\textrm{K}$. The dynamics of the carbon atoms above the melting points (${1273}\,\textrm{K}$ and ${1473}\,\textrm{K}$) are well activated, and the carbon atoms are freely exchanged in their positions. The average velocity of the carbon in the direction normal to the surface was estimated using the following expression:

1 $$\begin{aligned} {\bar{v}}=\sum _{t={50}}^{{100}\,\textrm{ns}}|z(t+\Delta t)-z(t)|/\Delta t \end{aligned}$$

The average velocities were ${5.4}\,{\textrm{mm}/\textrm{s}}$ at ${1073}\,\textrm{K}$, ${126}\,{\textrm{mm}/\textrm{s}}$ at ${1273}\,\textrm{K}$, and ${143}\,{\textrm{mm}/\textrm{s}}$ at ${1473}\,\textrm{K}$, and all velocities were much higher than the reported velocities of CNT growth. Therefore, we can conclude that the bottleneck process of CNT growth is not the carbon supply from inside the cementite structure, but the C–C bond formation at the interface. The pulled-up speed for CNT growth in this study was ${1}\,{\textrm{mm}/\textrm{s}}$, which is relatively fast with respect to the experimentally reported speed, but quite a possible speed from the viewpoint of carbon sources.

**Figure 3:**
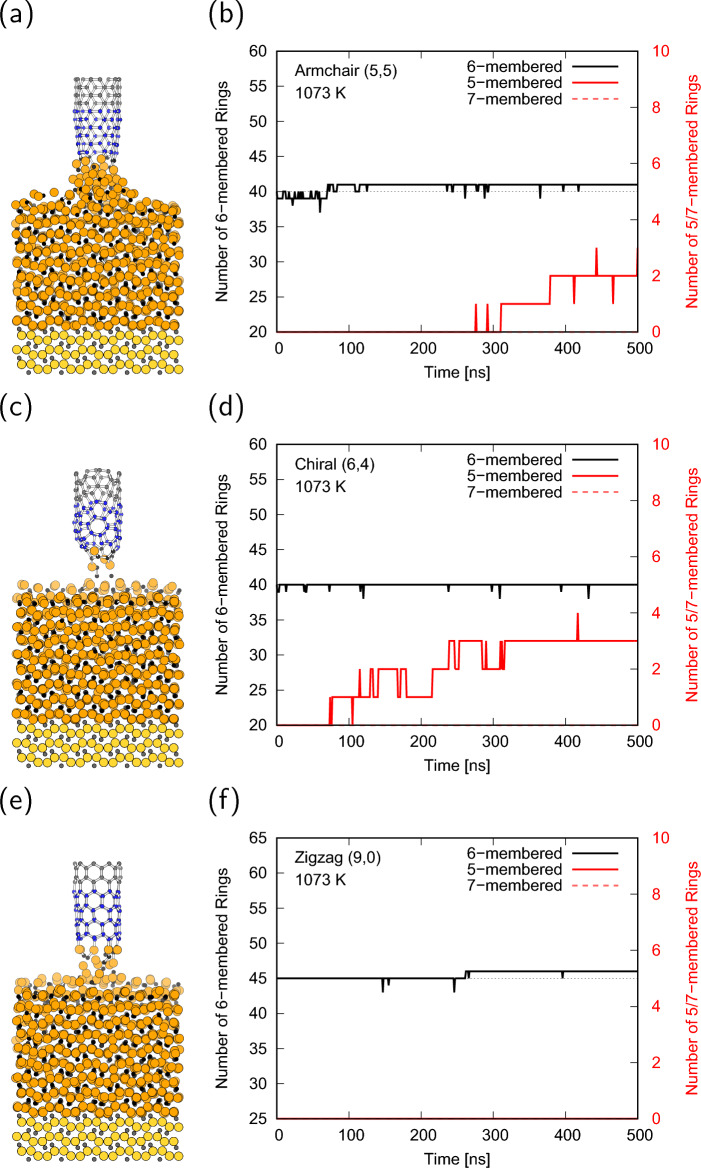
(**a**, **c**, **e**) MD snapshots after ${500}\,\textrm{ns}$ simulation, (**b**, **d**, **f**) number of 6-membered, 5-membered, and 7-membered rings of the (**a**, **b**) armchair, (**c**, **d**) chiral, and (**e**, **f**) zigzag CNTs at ${1073}\,\textrm{K}$. The dotted lines in (**b**, **d**, **f**) denote the initial number of 6-membered rings.

**Figure 4:**
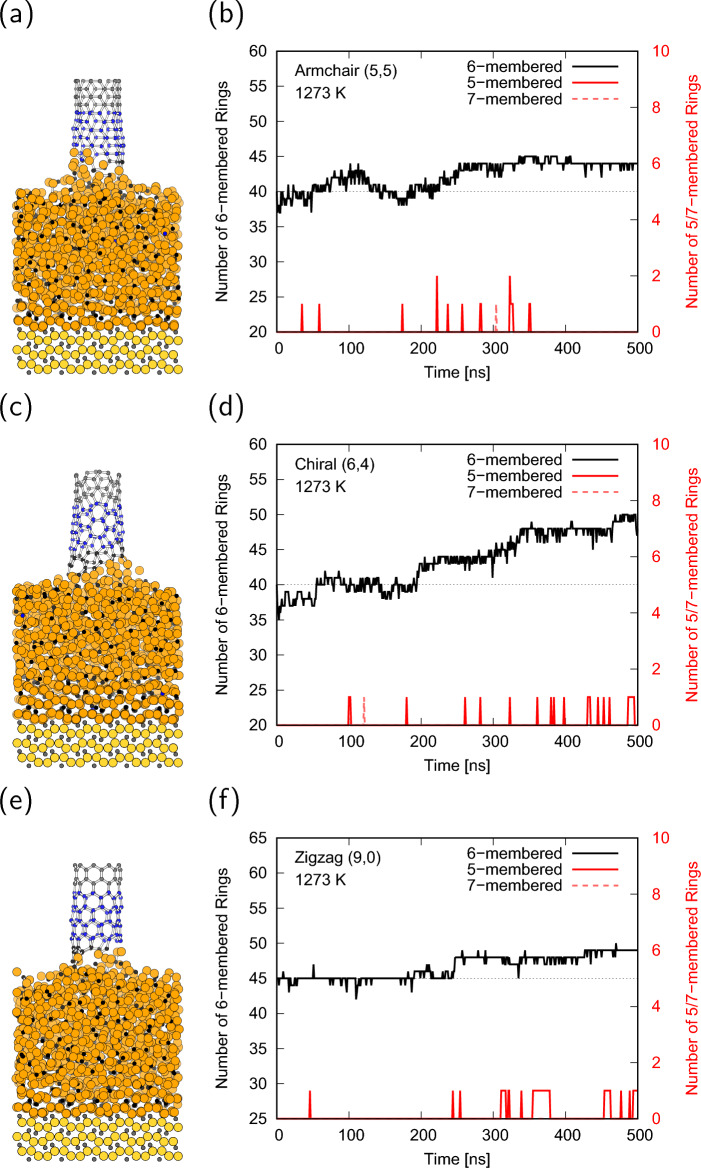
(**a**, **c**, **e**) MD snapshots after ${500}\,\textrm{ns}$ simulation, (**b**, **d**, **f**) number of 6-membered, 5-membered, and 7-membered rings of the (**a**, **b**) armchair, (**c**, **d**) chiral, and (**e**, **f**) zigzag CNTs at ${1273}\,\textrm{K}$. The dotted lines in (**b**, **d**, **f**) denote the initial number of 6-membered rings.

**Figure 5:**
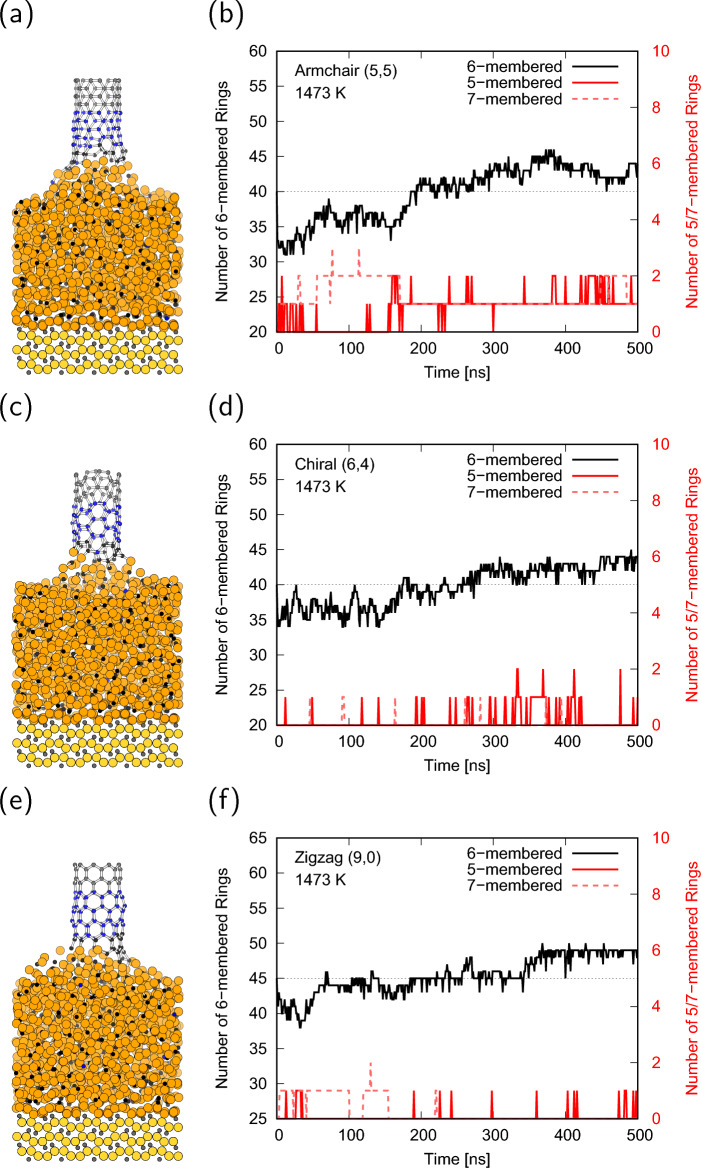
(**a**, **c**, **e**) MD snapshots after ${500}\,\textrm{ns}$ simulation, (**b**, **d**, **f**) number of 6-membered, 5-membered, and 7-membered rings of the (**a**, **b**) armchair, (**c**, **d**) chiral, and (**e**, **f**) zigzag CNTs at ${1473}\,\textrm{K}$. The dotted lines in (**b**, **d**, **f**) denote the initial number of 6-membered rings.

Based on the above results, MD simulations of the growing CNT on the cementite surface were performed at a pull-up speed of ${1}\,{\textrm{mm}/\textrm{s}}$. Full trajectories during 500 ns simulations are available in Supplementary Video S1-S3. Figure 3a,c,e shows the final structures of the ${500}\,{\textrm{ns}}$ MD simulations for armchair, chiral, and zigzag CNTs at ${1073}\,\textrm{K}$. This shows that the carbon supply from the cementite at ${1073}\,\textrm{K}$ is insufficient, and the pulled-up CNTs are separated from the surface of cementite for all chiralities. Although the average velocity of carbon atoms in cementite was faster than the CNT pulled-up speed, only carbon atoms near the surface moved, and carbon diffusion from the bottom of cementite did not occur. Most of the atoms in the cementite maintained their initial positions, and the carbon atoms in the cementite were unable to catch up with the pull-up velocity of the CNT, resulting in the lifting of the cementite for the armchair CNT and the breaking of the connections between CNT and cementite for the chiral and zigzag CNTs. The trajectories of the number of 6-membered rings were nearly unchanged regardless of the CNT chirality, as shown in Fig. 3b,d,f, whereas those of the 5-membered rings increase with simulation time because dangling bonds of the CNT edge are formed and tend to form bonds with neighbor carbon to reduce the energy. In contrast to the low-temperature condition, the final structures at ${1273}\,\textrm{K}$ indicate that the CNTs are connected to the cementite surface for all chiralities as shown in Fig. 4a,c,e. The number of 6-membered rings nearly monotonically increased from the initial value as the simulation time increased, and no stable defect rings, such as 5-membered or 7-membered rings were formed (Fig. 4b,d,f). Note that the increasing behavior of the 6-membered rings for the chiral CNT is different from that of the others at ${1273}\,\textrm{K}$. The number of 6-membered rings for the chiral CNT continued to increase throughout the simulation, whereas those for the armchair and zigzag CNTs were almost saturated at approximately half of the simulation time and only reached half of the numbers for chiral CNT. These results indicate that the chiral CNT can grow stably even at a speed of ${1}\,{\textrm{mm}/\textrm{s}}$ at ${1273}\,\textrm{K}$, which is above the melting point of cementite. Under high-temperature conditions, although CNTs eventually elongate from initial length as shown in Fig. 5a,c,e, the CNT were absorbed onto the cementite surface, and the reconstruction of the CNT occurred in the initial stage of the simulations. Therefore, the number of 6-membered rings alternately decreases at the initial stage, increases below the initial value with the undulation of cementite, and finally increases over the initial value, as shown in Fig. 5b,d,f. The reconstruction of the defect rings during the CNT reconstruction process is shown in Fig. 5b. There is a possibility of defects owing to the high fluidity of cementite at ${1473}\,\textrm{K}$. Temperatures above the melting point are required for CNT growth at a speed of ${1}\,\textrm{mm}/\textrm{s}$, but too high temperature may cause defects, which change chirality or cause CNTs to separate from cementite surface.

**Figure 6:**
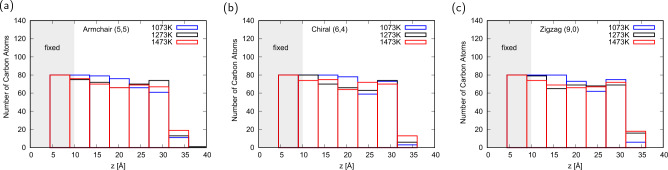
Distribution of the carbon atoms in cementite after a ${500}\,\textrm{ns}$ simulation of (**a**) armchair, (**b**) chiral, and (**c**) zigzag CNTs.

To investigate the carbon diffusion in cementite, we analyzed the change in the z position of the carbon atoms in cementite over the simulation time. At ${1073}\,\textrm{K}$, only the carbon atoms at a depth of approximately ${1}\,\textrm{nm}$ from the surface moved, and most of the carbon atoms in cementite almost maintained their initial position, as mentioned above. However, the carbon atoms moved over the entire movable area with a thickness of ${2}\,\textrm{nm}$ at ${1273}\,\textrm{K}$ and ${1473}\,\textrm{K}$ because of the melting of cementite. Reflecting this difference in carbon diffusion, the distribution of carbon atoms after the ${500}\,\textrm{ns}$ simulation also showed different behavior between ${1073}\,\textrm{K}$ and above, as shown in Fig. 6. Regardless of CNT chirality, the number of carbon atoms decreased only near the surface at ${1073}\,\textrm{K}$, whereas the carbon distributions at ${1273}\,\textrm{K}$ and ${1473}\,\textrm{K}$ were flatter than those at ${1073}\,\textrm{K}$. At ${1273}\,\textrm{K}$ and ${1473}\,\textrm{K}$, carbon atoms are supplied from the lower part of the cementite to the cementite surface, where carbon atoms have been reduced due to being used for CNT growth. Because more carbon atoms in cementite can contribute to CNT growth than those at ${1073}\,\textrm{K}$, CNT can grow at ${1273}\,\textrm{K}$ and ${1473}\,\textrm{K}$ at a CNT pull-up velocity of ${1}\,\textrm{mm}/\textrm{s}$; in particular, chiral CNT can continue to grow.

**Figure 7:**
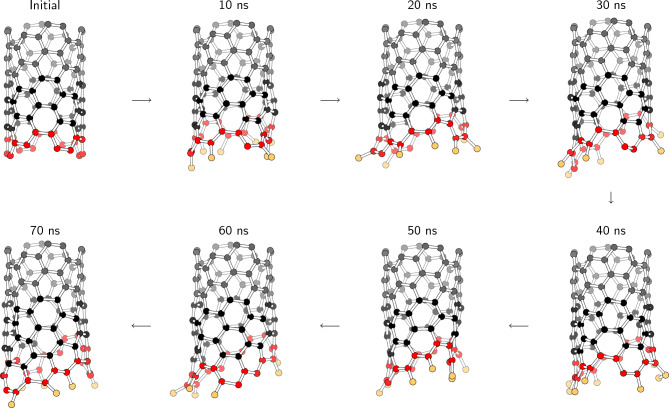
MD snapshots of the initial growth step of the chiral CNT at ${1273}\,\textrm{K}$. Black, gray, red, and orange atoms denote the normal carbon atoms, carbon atoms pulled up by a constant velocity, CNT edges, and carbon atoms attached to the CNT edges, respectively.

Figure 7 shows the initial growth steps of chiral CNT. New 6-membered rings were obliquely constructed along the CNT edges. This indicates an apparent chiral growth, leading to a chiral (6,4) CNT, which is a near-armchair CNT and is easier to grow compared to pure armchair and zigzag CNTs, as reported by Artyukhov et al.^39^. The edges of the armchair and zigzag CNTs are parallel to the surface of the cementite structure; therefore, many carbon atoms should approach from the cementite to the CNT edge simultaneously for efficient CNT growth. However, the carbon atoms at the edge of the chiral CNT are not in the same plane, and thus a time lag for reaching the carbon atoms is permitted. Therefore, chiral CNT continue to grow stably at a speed of ${1}\,\textrm{mm}/\textrm{s}$ at ${1273}\,\textrm{K}$, whereas the growth of the armchair and zigzag CNTs cannot completely catch up with the CNT pull-up velocity.

## Conclusions

We performed MD simulations of the CNT growth process under the FCCVD method. Melting-point depression and tensile strain, which are features of the FCCVD method, were considered. Our simulation demonstrated that the carbon atoms in the cementite structure over its melting point diffuse well, and their speed is faster than ${1}\,\textrm{mm}/\textrm{s}$, which is the fastest speed of CNT growth in the FCCVD method. The CNT could not grow at ${1073}\,\textrm{K}$ because of the insufficient supply of carbon, and could grow with many defect rings at ${1473}\,\textrm{K}$ because iron atoms from cementite structure devoured a part of CNT structure. The number of 6-membered rings nearly monotonically increased in the chiral (6,4) CNT at ${1273}\,\textrm{K}$, whereas it was stagnant in the armchair (5,5) and zigzag (9,0) CNTs.

### Supplementary Information

Supplementary Legends.

Supplementary Video S1.

Supplementary Video S2.

Supplementary Video S3.

## Data Availability

The datasets generated during and/or analyzed during the current study are available from the corresponding author on reasonable request.

## References

[1] Dresselhaus MS, Dresselhaus G, Eklund PC (1996). Science of Fullerenes and Carbon Nanotubes.

[2] White CT, Mintmire JW (2005). Fundamental properties of single-wall carbon nanotubes. J. Phys. Chem. B.

[3] Kataura H (1999). Optical properties of single-wall carbon nanotubes. Synth. Met..

[4] Ruoff RS, Qian D, Liu WK (2003). Mechanical properties of carbon nanotubes: Theoretical predictions and experimental measurements. C. R. Phys..

[5] Oberlin A, Endo M, Koyama T (1976). Filamentous growth of carbon through benzene decomposition. J. Cryst. Growth.

[6] Iijima S (1991). Helical microtubules of graphitic carbon. Nature.

[7] Bai Y (2018). Carbon nanotube bundles with tensile strength over 80 GPa. Nat. Nanotechnol..

[8] Barkia B (2020). On the origin of the high tensile strength and ductility of additively manufactured 316L stainless steel: Multiscale investigation. Sci. Technol..

[9] Han Z, Fina A (2011). Thermal conductivity of carbon nanotubes and their polymer nanocomposites: A review. Prog. Polym. Sci..

[10] Thess A (1996). Crystalline ropes of metallic carbon nanotubes. Science.

[11] Journet C (1997). Large-scale production of single-walled carbon nanotubes by the electric-arc technique. Nature.

[12] Endo M (1988). Grow carbon fibers in the vapor phase. Chem. Tech..

[13] Li WZ (1996). Large-scale synthesis of aligned carbon nanotubes. Science.

[14] Cassell AM, Raymakers JA, Kong J, Dai H (1999). Large scale CVD synthesis of single-walled carbon nanotubes. J. Phys. Chem. B.

[15] Huang S, Woodson M, Smalley R, Liu J (2004). Growth mechanism of oriented long single walled carbon nanotubes using “fast-heating” chemical vapor deposition process. Nano Lett..

[16] Atiyah MR (2011). Low temperature growth of vertically aligned carbon nanotubes via floating catalyst chemical vapor deposition method. J. Mater. Sci. Technol..

[17] Li Y-J, Ma C, Kang J-L, Shi J-L, Shi Q, Wu D-H (2017). Preparation of diameter-controlled multi-wall carbon nanotubes by an improved floating-catalyst chemical vapor deposition method. New Carbon Mater..

[18] Kinoshita T, Karita M, Nakano T, Inoue Y (2019). Two step floating catalyst chemical vapor deposition including in situ fabrication of catalyst nanoparticles and carbon nanotube forest growth with low impurity level. Carbon.

[19] Dong L (2020). Continuous synthesis of double-walled carbon nanotubes with water-assisted floating catalyst chemical vapor deposition. Nanomaterials.

[20] Hou P-X (2022). Synthesis of carbon nanotubes by floating catalyst chemical vapor deposition and their applications. Adv. Funct. Mater..

[21] Fujimori T (2022). One step fabrication of aligned carbon nanotubes using gas rectifier. Sci. Rep..

[22] Chen DR, Chitranshi M, Schulz M, Shanov V (2019). A review of three major factors controlling carbon nanotubes synthesis from the floating catalyst chemical vapor deposition. Nano Life.

[23] Zhu Z (2019). Rate-selected growth of ultrapure semiconducting carbon nanotube arrays. Nat. Commun..

[24] Motta MS, Moisala A, Kinloch IA, Windle AH (2008). The role of sulphur in the synthesis of carbon nanotubes by chemical vapour deposition at high temperatures. J. Nanosci. Nanotechnol..

[25] Wirth CT, Zhang C, Zhong G, Hofmann S, Robertson J (2009). Diffusion- and reaction-limited growth of carbon nanotube forests. ACS Nano.

[26] Matsumoto N (2018). One millimeter per minute growth rates for single wall carbon nanotube forests enabled by porous metal substrates. RSC Adv..

[27] Irle S (2009). Milestones in molecular dynamics simulations of single-walled carbon nanotube formation: A brief critical review. Nano Res..

[28] Raji K, Sobhan CB (2013). Simulation and modeling of carbon nanotube synthesis: Current trends and investigations. Nanotechnol. Rev..

[29] Henriksson KOE, Nordlund K (2009). Simulations of cementite: An analytical potential for the Fe–C system. Phys. Rev. B.

[30] Nosé S (1984). A molecular dynamics method for simulations in the canonical ensemble. Mol. Phys..

[31] Hoover WG (1985). Canonical dynamics: Equilibrium phase-space distributions. Phys. Rev. A.

[32] Shinoda W, Shiga M, Mikami M (2004). Rapid estimation of elastic constants by molecular dynamics simulation under constant stress. Phys. Rev. B.

[33] Plimpton S (1995). Fast parallel algorithms for short-range molecular dynamics. J. Comput. Phys..

[34] Lau TT (2007). Many-body potential for point defect clusters in Fe–C alloys. Phys. Rev. Lett..

[35] Ruda M, Farkas D, Garcia G (2009). Atomistic simulations in the Fe–C system. Comput. Mater. Sci..

[36] Liyanage LSI (2014). Structural, elastic, and thermal properties of cementite (Fe_3_C) calculated using a modified embedded atom method. Phys. Rev. B.

[37] Lebedeva IV, Minkin AS, Popov AM, Knizhnik AA (2019). Elastic constants of graphene: Comparison of empirical potentials and DFT calculations. Phys. E Low-Dimens. Syst. Nanostruct..

[38] Brenner DW (1990). Empirical potential for hydrocarbons for use in simulating the chemical vapor deposition of diamond films. Phys. Rev. B.

[39] Artyukhov VI, Penev ES, Yakobson BI (2014). Why nanotubes grow chiral. Nat. Commun..

